# National survey of colistin resistance among carbapenemase-producing *Enterobacteriaceae* and outbreak caused by colistin-resistant OXA-48-producing *Klebsiella pneumoniae*, France, 2014

**DOI:** 10.2807/1560-7917.ES.2016.21.37.30339

**Published:** 2016-09-15

**Authors:** Aurélie Jayol, Laurent Poirel, Laurent Dortet, Patrice Nordmann

**Affiliations:** 1 Emerging Antibiotic Resistance Unit, Medical and Molecular Microbiology, Department of Medicine, University of Fribourg, Fribourg, Switzerland; 2 Associated National Reference Centre for Antibiotic Resistance, Le Kremlin-Bicêtre, France; 3 Faculty of Medicine, South-Paris University, Le Kremlin-Bicêtre, France; 4 Bacteriology-Hygiene unit, Hospital Bicêtre, Assistance Publique /Hôpitaux de Paris, Le Kremlin-Bicêtre, France; 5 University of Lausanne and University Hospital Center, Lausanne, Switzerland

**Keywords:** colistin, polymyxins, resistance, *Klebsiella pneumoniae*, *Enterobacter cloacae*, outbreak

## Abstract

From January 2014 to December 2014, 972 consecutive non-replicate carbapenemase-producing *Enterobacteriaceae* isolates from colonised or infected patients were collected at the Associated French National Reference Centre as part of the French national survey on antimicrobial resistance. It included 577 *Klebsiella* spp. (59%), 236 *Escherichia coli* (24%), 108 *Enterobacter* spp. (11%), 50 *Citrobacter* spp. (5%), and a single *Salmonella* spp. isolate (0.1%). Of 561 *K. pneumoniae* isolates, 35 were found to be resistant to colistin (6.2%). PFGE analysis revealed a clonal outbreak involving 15 *K. pneumoniae* isolates belonging to sequence type ST11, recovered in a single hospital in the Picardie region in northern France. Those clonally related isolates showed variable levels of resistance to colistin, ranging from 4 to 64 mg/L. They harboured the *bla*_OXA-48_ carbapenemase gene and the *bla*_CTX-M-15_ extended-spectrum beta-lactamase gene. Among the 91 *Enterobacter cloacae* isolates, seven were resistant to colistin and produced different types of carbapenemases. Surprisingly, none of the *E. coli* and *Citrobacter* spp. isolates showed resistance to colistin. This national survey including carbapenemase-producing isolates recovered in 2014 reported a high rate of colistin resistance in *K. pneumoniae* and *E. cloacae* (6.2% and 7.7%, respectively) in France.

## Introduction

Carbapenemase-producing *Enterobacteriaceae* (CPE) resistant to colistin are increasingly reported. They represent an additional link in the development of pan-drug resistance. However, the epidemiology of colistin resistance among enterobacterial isolates is currently almost unknown in most parts of the world. In Italy, an increase in carbapenemase-producing *Enterobacteriaceae* has been noted in the past years, but the situation remains unknown in France [1]. The lack of information about the prevalence of colistin resistance among multidrug-resistant enterobacterial isolates derives from several reasons: (i) so far, there has been limited interest in that field, (ii) methods used for determination of colistin susceptibility are not adequate, and (iii) the lack of well-defined breakpoints does not allow precise determination of prevalence. However, the recent identification of a plasmid-borne polymyxin resistance determinant (MCR-1) raised a very serious concern in that resistance to colistin might widely disseminate [2].

The aim of this study was to evaluate retrospectively the prevalence of colistin resistance among a collection of CPE strains recovered in France during a period of one year and to analyse the phenotypic, genotypic features and clonality of the colistin-resistant isolates.

## Methods

### Carbapenemase-producing *Enterobacteriaceae* isolates

From January to December 2014, 972 consecutive non-duplicate isolates of carbapenemase-producing *Enterobacteriaceae* were isolated in private laboratories and hospitals in France either by screening for colonisation or by analysing clinical samples in the context of infections. They were recovered from rectal swabs or stools (n = 625), urine samples (n = 250), respiratory tract samples (n = 35), blood samples (n = 22), wounds (n = 24), catheter (n = 7), vaginal swabs (n = 3) and other specimens (n = 6). Those isolates were sent to the Associated French National Reference Centre for characterisation of resistance mechanisms to carbapenems as part of the French antibiotic resistance survey. The 972 carbapenemase-producing *Enterobacteriaceae* isolates included 577 isolates of *Klebsiella* spp. (59%), 236 isolates of *Escherichia coli* (24%), 108 isolates of *Enterobacter* spp. (11%), 50 isolates of *Citrobacter* spp. (5%), and a single isolate of *Salmonella* spp. (0.1%). Species that are naturally resistant to colistin (*Proteus* spp., *Morganella morganii*, *Providencia* spp., and *Serratia* spp.) had been excluded before the initiation of this study. Only a single isolate per patient was included in the study. All isolates were identified using the Microflex bench-top MALDI-TOF mass spectrometer (Bruker, Champs-sur-Marne, France).

### Antimicrobial susceptibility testing

Minimum inhibitory concentrations (MIC) of colistin (CS) were determined using broth microdilution method according to the guidelines of the Clinical Laboratory Standards Institute (CLSI) [3]. As recommended, *E. coli* ATCC 25922 was used as quality control strain.

For the colistin-resistant isolates, susceptibility to other classes of antibiotics was also tested. Susceptibility to imipenem, ertapenem, and tigecycline was tested by broth microdilution method according to CLSI guidelines, whereas susceptibility to the other antibiotics was tested by the standardised agar disk diffusion method according to the guidelines of the European Committee on Antimicrobial Susceptibility Testing (EUCAST) [4]. The antibiotics tested using disk diffusion method were: amoxicillin (AMX), amoxicillin/clavulanic acid (AMC), cefotaxime (CTX), cefoxitin (FOX), ceftazidime (CAZ), cefepime (FEP), temocillin (TEM), ciprofloxacin (CIP), gentamicin (GM), amikacin (AK), trimethoprim-sulfamethoxazole (SXT) and fosfomycin (FOS).

The MIC results for colistin and the disk diffusion diameters were interpreted according to susceptibility breakpoints of the European Committee on Antimicrobial Susceptibility Testing (EUCAST) [4].

### Molecular characterisation

The *mgrB* genes of *K. pneumoniae* and *Enterobacter* spp. isolates were amplified using specific primers (Table 1), knowing that the MgrB protein is a negative regulator of the PhoPQ two-component system and that alterations in the *mgrB* gene are commonly involved in acquisition of colistin resistance in *K. pneumoniae* [5-7]. The plasmid-mediated *mcr-1* gene encoding colistin resistance was sought as described previously [2]. Detection of extended-spectrum beta-lactamases (ESBL) and carbapenemases genes was performed with specific primers as described previously [8]. Both strands of the amplification products obtained were sequenced with an ABI 3100 sequencer (Applied Biosystems, Foster City, US). The nucleotide and deduced protein sequences were analysed at the National Centre for Biotechnology Information website (www.ncbi.nlm.nih.gov) by the Basic Local Alignment Search Tool (BLAST) programme.

**Table 1:** Oligonucleotides used as primers in this study, France, January–December 2014

Oligonucleotides	Sequence (5’-3’)	Reference
*Kpn mgrB* ext F	TTA AGA AGG CCG TGC TAT CC	[7]
*Kpn mgrB* ext R	AAG GCG TTC ATT CTA CCA CC	[7]
*Kpn mgrB* int F	CGG TGG GTT TTA CTG ATA GTC	This study
*Kpn mgrB* int R	GAA CAT CCT GGT CGC ACA TT	This study
*Ent mgrB* ext F	CGG TTT ACT CTA TGA AAC AAG TGC	This study
*Ent mgrB* ext R	GCG AAG GAA GGA AAT CAC CT	This study

### Genotyping

Genotyping was performed to evaluate the clonal relationship of the colistin-resistant *K. pneumoniae* and *E. cloacae* isolates by pulsed-field gel elctrophoresis (PFGE) with *Xba*I-digested genomic DNA and interpreted according to Tenover criteria [9]. Multilocus sequence typing (MLST) for *K. pneumoniae* was performed using the simplified protocol at the Institut Pasteur website (http://bigsdb.pasteur.fr/klebsiella/klebsiella.html) [10].

## Results

### *Klebsiella pneumoniae*

**Figure 1:**
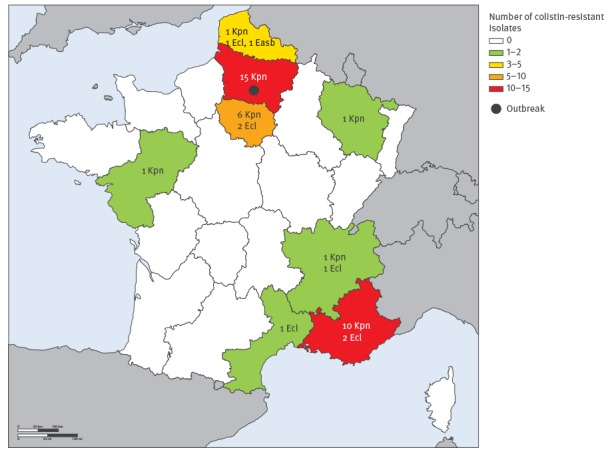
Geographic distribution of colistin-resistant *Enterobacteriaceae* isolates, France, January–December 2014 (n = 43)

**Figure 2:**
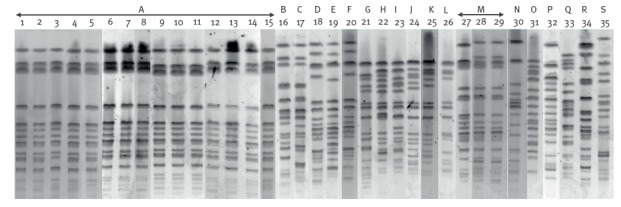
PFGE patterns of *Xba*I-digested chromosomal DNA of colistin-resistant *Klebsiella pneumoniae* isolates, France, January–December 2014 (n = 35)

Of 561 *K. pneumoniae* isolates, 35 were found to be resistant to colistin (6%). Fifteen of the 35 colistin-resistant *K. pneumoniae* isolates were recovered from a single hospital in the Picardie region, northern France (Figure 1). We could not obtain the exact dates of their isolations due to the retrospective nature of the study. These isolates had mostly been recovered from rectal swab specimens, but also from a catheter, a urinary sample, a wound exudate and a respiratory specimen (isolates 1 to 15, Table 2). PFGE analysis revealed that the 15 isolates were clonally related (Figure 2, Table 2). The clone was of the ST11 type, and was susceptible only to cefoxitin, amikacin and fosfomycin (Table 2). A single isolate among these 15 was susceptible to tigecycline. The 15 isolates harboured both the *bla*_OXA-48_ carbapenemase gene, and the *bla*_CTX-M-15_ extended-spectrum beta-lactamase (ESBL) gene, and the MICs for colistin ranged from 4 to 64 mg/L (Table 2).

Easb: *Enterobacter asburiae*; Ecl: *Enterobacter cloacae*; Kpn: *Klebsiella pneumoniae.*

**Table 2:** Characteristics of the colistin-resistant *Klebsiella pneumoniae* and *Enterobacter* spp. clinical isolates, France, January–December 2014 (n = 43)

Isolate	Site of isolation	Origin	MIC CS^a^	*mgrB* genotype	Carbapenemase	Associated beta-lactamase	Co-resistances^b^	ST	PFGE
1	?	Picardie	32	*mgrB* WT	OXA-48	CTX-M-15	CIP GM SXT	11	A
2	Catheter	Picardie	8	*mgrB* WT	OXA-48	CTX-M-15	CIP GM SXT TIG	11	A
3	Rectal swab	Picardie	4	*mgrB* WT	OXA-48	CTX-M-15	CIP GM SXT TIG	11	A
4	Rectal swab	Picardie	4	*mgrB* WT	OXA-48	CTX-M-15	CIP GM SXT TIG	11	A
5	Rectal swab	Picardie	64	*mgrB* WT	OXA-48	CTX-M-15	CIP GM SXT TIG	11	A
6	Urine	Picardie	32	*mgrB* WT	OXA-48	CTX-M-15	CIP GM SXT TIG	11	A
7	Rectal swab	Picardie	64	*mgrB* WT	OXA-48	CTX-M-15	CIP GM SXT TIG	11	A
8	Wound	Picardie	4	*mgrB* WT	OXA-48	CTX-M-15	CIP GM SXT TIG	11	A
9	Respiratory	Picardie	4	*mgrB* WT	OXA-48	CTX-M-15	CIP GM SXT TIG	11	A
10	Rectal swab	Picardie	4	*mgrB* WT	OXA-48	CTX-M-15	CIP GM SXT TIG	11	A
11	Rectal swab	Picardie	8	*mgrB* WT	OXA-48	CTX-M-15	CIP GM SXT TIG	11	A
12	Rectal swab	Picardie	64	*mgrB* WT	OXA-48	CTX-M-15	CIP GM SXT TIG	11	A
13	Rectal swab	Picardie	8	*mgrB* WT	OXA-48	CTX-M-15	CIP GM SXT TIG	11	A
14	Rectal swab	Picardie	4	*mgrB* WT	OXA-48	CTX-M-15	CIP GM SXT TIG	11	A
15	Rectal swab	Picardie	4	*mgrB* WT	OXA-48	CTX-M-15	CIP GM SXT TIG	11	A
16	Rectal swab	Nord-Pas-de-Calais	128	IS*1R* in promoter region (between nt −45 and −46)	OXA-48	CTX-M-15	CIP GM SXT TIG	147	B
17	Rectal swab	Ile-de-France	128	*mgrB* WT	NDM	CTX-M-15	CIP GM SXT TIG	147	C
18	Rectal swab	PACA	>128	IS*Kpn26*-like in coding region (between nt +74 and +75)	KPC	-	CIP AK SXT TIG	258	D
19	Rectal swab	Rhône-Alpes	128	MgrB truncated (27 amino acids)	KPC	-	CIP AK SXT TIG	258	E
20	Blood	PACA	16	Full gene deletion^c^	KPC	-	CIP AK SXT TIG	258	F
21	Rectal swab	Lorraine	64	Single nucleotide deletion (nt 74)	OXA-48	CTX-M-15	CIP GM	101	G
22	Rectal swab	Ile-de-France	32	Single nucleotide deletion (nt 23)	OXA-48	CTX-M-15	CIP GM	101	H
23	Rectal swab	Ile-de-France	64	IS*1R* in promoter region (between nt −36 and −37)	OXA-48 + NDM	CTX-M-15	CIP GM	101	I
24	Urine	PACA	64	IS*1R* in promoter region (between nt −45 and −46)	OXA-48	CTX-M-15	CIP GM SXT TIG	101	J
25	Abcess	Ile-de-France	32	MgrB M27K	OXA-48	CTX-M-15	CIP GM	101	K
26	Urine	Pays de la Loire	128	Duplication of 19 nucleotides	OXA-48	CTX-M-15	CIP SXT FOS	101	L
27	Urine	PACA	64	IS*1R* in coding region (between nt +21 and +22)	OXA-48	CTX-M-15	CIP GM SXT TIG	307	M
28	Rectal swab	PACA	64	*mgrB* WT	OXA-48	CTX-M-15	CIP GM SXT TIG FOS	307	M
29	Rectal swab	PACA	64	IS*5*-like in coding region (between nt +74 and +75)	OXA-48	CTX-M-15	CIP GM SXT TIG	307	M
30	Urine	PACA	>128	Full gene deletion^b^	OXA-48	-	CIP GM AK SXT TIG FOS	611	N
31	Rectal swab	Ile-de-France	32	IS*Kpn14*-like in promoter region (between nt −45 and −46)	OXA-48	-	CIP GM SXT TIG	23	O
32	Rectal swab	PACA	32	IS*102*-like in coding region (between nt +36 and +37)	OXA-48	CTX-M-15	CIP GM SXT TIG	20	P
33	Respiratory	Ile-de-France	32	IS*1R* in promoter region (between nt −61 and −62)	OXA-48	CTX-M-15	CIP SXT TIG FOS		Q
34	Blood	PACA	>128	*mgrB* WT	OXA-48	CTX-M-15	CIP SXT TIG	39	R
35	Rectal swab	PACA	32	MgrB truncated (32 amino acids)	OXA-48	-	FOS	13	S
36	Rectal swab	Nord-Pas-de-Calais	64	*mgrB* WT	OXA-48 + VIM	CTX-M-15	CIP GM SXT	NA	T
37	Stools	Languedoc-Roussillon	64	*mgrB* WT	OXA-48	CTX-M-15	GM AK	NA	U
38	Respiratory	Ile-de-France	32	*mgrB* WT	VIM	-	SXT TIG	NA	V
39	Rectal swab	PACA	>128	*mgrB* WT	OXA-48	-	CIP GM SXT TIG	NA	W
40	Rectal swab	Ile-de-France	16	*mgrB* WT	OXA-48	-	FOS	NA	X
41	Rectal swab	PACA	>128	*mgrB* WT	IMP	CTX-M-2	No	NA	Y
42	Respiratory	Rhône-Alpes	>128	*mgrB* WT	OXA-48	-	TIG	NA	Z
43	Urine	Nord-Pas-de-Calais	>128	*mgrB* WT	VIM-1	-	CIP TIG	NA	α

Isolates 1-35: *Klebsiella pneumoniae*; 36–42: *Enterobacter* cloacae; 43: *Enterobacter asburiae.*

AK: amikacin; CIP: ciprofloxacin; CS: colistin; FOS: fosfomycin; GM: gentamicin; MIC: minimum inhibitory concentration; NA: not applicable; nt: nucleotide; PACA: Provence-Alpes-Côte-d’Azur; PFGE: pulsed-field gel electrophoresis; ST: sequence type; SXT: trimethoprim-sulfamethoxazole; TIG: tigecycline; WT: wildtype.

^a^ MIC of colistin determined by broth microdilution method.

^b^ Resistant or intermediate susceptibility to antibiotic.

^c^ Full gene deletion: no PCR product was detected with external or internal primers.

The numbers correspond to the isolates from Table 2 and the letters indicate the PFGE type.

The other 20 colistin-resistant *K. pneumoniae* strains were mostly recovered from the regions Ile-de-France (n = 6) and Provence-Alpes-Côte d’Azur (n = 10) (Figure 1). These strains presented high MIC values for colistin ranging from 16 to >128 mg/L (isolates 16 to 35, Table 2). They produced either the carbapenemases OXA-48 (15/20), KPC-2 (3/20), NDM-1 (1/20), or both OXA-48 and NDM-1 together (1/20) (Table 2). Overall, 14 of the 20 isolates produced the ESBL CTX-M-15. PFGE analysis identified 18 clonal patterns among the 20 isolates (n = 3 for clone M) (Figure 2, Table 2), and MLST assigned the isolates to eight sequence types (STs) (Table 2).

Sequencing of the *mgrB* gene of those *K. pneumoniae* isolates revealed various *mgrB* alterations and none of the strains harboured the plasmid-encoded *mcr-1* gene.

**Figure 3:**
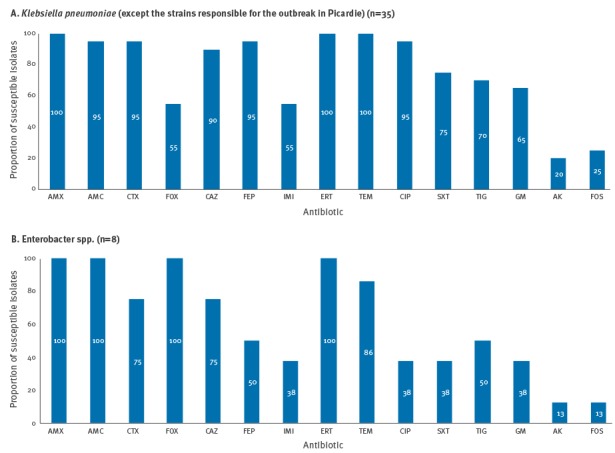
Antimicrobial non-susceptibilities among colistin-resistant isolates, France, January–December 2014

AK: amikacin; AMC: amoxicillin/clavulanic acid; AMX: amoxicillin; CAZ: ceftazidime; CIP: ciprofloxacin; CTX: cefotaxime; ERT: ertapenem; FEP: cefepime; FOS: fosfomycin; FOX: cefoxitin; GM: gentamicin; IMI: imipenem; SXT: trimethoprim-sulfamethoxazole; TEM: temocillin; TIG: tigecycline.

Antimicrobial susceptibility data for the colistin-resistant *K. pneumoniae* isolates not involved in the outbreak revealed that most isolates (19/20) were non-susceptible to third- and fourth-generation cephalosporins (Figure 3A). They were also frequently resistant to ciprofloxacin (19/20), trimethoprim-sulfamethoxazole (15/20) and tigecycline (14/20). They were less often resistant to gentamicin and cefoxitin (13/20 and 11/20, respectively). Amikacin and fosfomycin remained the most active agents against colistin-resistant *K. pneumoniae* (16/20 and 15/20 were susceptible, respectively) (Figure 3A).

### *Enterobacter* spp.

Among the 91 *Enterobacter cloacae* isolates, seven were resistant to colistin (7.7%). They showed high MIC values for colistin (ranging from 16 to >128 mg/L) (isolates 36 to 42, Table 2). They produced the carbapenemases OXA-48 (4/7), VIM-1 (1/7), IMP-1 (1/7), or both OXA-48 and VIM-1 together (1/7) (Table 2). In total, three of seven strains were CTX-M producers, with two isolates producing CTX-M-15 and a single isolate producing CTX-M-2. The colistin-resistant *E. cloacae* isolates were recovered in different geographical regions in France (Figure 1, Table 2), and results of the PFGE analysis revealed that they were not clonally related (data not shown).

The single carbapenem-resistant *E. asburiae* strain was resistant to colistin. It had an MIC of colistin above 128 mg/L and produced the VIM-1 carbapenemase (isolate 43, Table 2).

All *Enterobacter* spp. isolates had a wild-type *mgrB* gene, leaving unexplained the colistin resistance mechanism (*E. cloacae* and *E. asburiae*) (Table 2).

Of the eight colistin-resistant *Enterobacter spp*. isolates, four were non-susceptible to cefepime and tigecycline, and three were non-susceptible to ciprofloxacin, trimethoprim-sulfamethoxazole and gentamicin (Figure 3B). Amikacin and fosfomycin were the most active agents against colistin-resistant *E. cloacae* (all seven isolates were susceptible) (Figure 3B).

### Other species

None of the *E. coli* (n = 236) and *Citrobacter* spp. (n = 50) isolates were resistant to colistin.

## Discussion

We describe here a clonal outbreak involving 15 *K. pneumoniae* isolates recovered from a single hospital in the Picardie region in northern France. This outbreak was caused by a colistin-resistant OXA-48 and CTX-M-15-producing *K. pneumoniae* of ST11 type that was susceptible only to cefoxitin, amikacin and fosfomycin. Surprisingly, those clonally related isolates had variable MIC values for colistin ranging from 4 to 64 mg/L. An ST11 clone co-producing OXA-48 and CTX-M-15 was responsible for a large outbreak involving 44 patients in a hospital in Madrid, Spain, from 2009 to 2014 but only 3.4% of the isolates were resistant to colistin [11].

Several outbreaks of colistin-resistant KPC-producing *K. pneumoniae* (mainly attributed to the international epidemic clone type ST258) have been reported across Europe, in Greece [12,13], Hungary [14], Italy [15-17] and the Netherlands [18]. A single outbreak of colistin-resistant VIM-1-producing *K. pneumoniae* has also been described in Spain [19]. 

We report also 20 colistin-resistant *K. pneumoniae* strains recovered from the regions Ile-de-France and Provence-Alpes-Côte d’Azur. These strains belonged to 10 sequence types (n = 2 ST147, n = 3 ST258, n = 6 ST101, n = 3 ST307) and PFGE analysis identified 18 patterns among the 20 isolates. All three KPC-producing *K. pneumoniae* isolates belonged to ST258, the most common clone for KPC-producing isolates [20]. The OXA-48-producing *K. pneumoniae* isolates belonged to nine sequence types with six strains that were ST101, the most common clone identified among OXA-48-positive *K. pneumoniae* [21].

Sequencing of the *mgrB* gene revealed *mgrB* alterations which are likely to be responsible for colistin resistance as described previously [5-7]. Interestingly, the three strains belonging to the single clone M recovered in the Provence-Alpes Côtes-d’Azur region had different mechanisms of *mgrB* inactivation (Table 2). The occurrence of such different mechanisms of colistin resistance among clonally related isolates indicates that it is not the product of clonal dissemination of a single colistin-resistant *K. pneumoniae* strain, but rather clonal dissemination of a carbapenemase-producing isolate, which has acquired colistin resistance thereafter.

The rates of colistin resistance among the carbapenemase-producing isolates were 7.7% for *Enterobacter* spp. and 3.6% for *K. pneumoniae* isolates (excluding the isolates responsible for the outbreak in the Picardie region). The resistance rate observed among the carbapenemase-producing *K. pneumoniae* isolates was much lower than the high rates reported in the neighbouring countries of southern Europe such as Spain (20%) [19] and Italy (43%) [1].

None of the 236 carbapenemase-producing *E. coli* isolates were colistin-resistant or carried the *mcr-1* gene. This is surprising considering that a recent report of the French antimicrobial resistance Resapath surveillance network identified the plasmid-borne *mcr-1* gene in 21% of ESBL-producing *E. coli* isolates recovered from faeces of veal calves in France between 2005 and mid-2014 [22]. The plasmid-borne *mcr-1* colistin resistance gene has also been found in many neighbouring countries of France, for example among ESBL-producing *Enterobacteriaceae* isolates recovered from river water and imported vegetable samples in Switzerland [23], in *E. coli* isolates recovered from calves and piglets in Belgium [24], in swine and human wound infections in Germany [25], and in food and human bloodstream infections in Denmark [26]. The *mcr-1* gene was also detected in *Salmonella enterica* from food samples in Portugal [27] and France [28]. An *E. coli* isolate co-harbouring the *bla*_VIM-1_ carbapenemase gene and the *mcr-1* gene was described in Switzerland [29] and an isolate co-producing NDM-9 and MCR-1 was reported from China [30]. We believe that the plasmid carrying the *mcr-1* gene might be currently more prevalent among ESBL-producing isolates than among carbapenemase-producing isolates in human samples, which would explain why we did not identify this gene in our collection of carbapenemase-producing isolates.

Amikacin and fosfomycin were most effective against the colistin and carbapenem-resistant *K. pneumoniae* (susceptibility rates of 80% and 75%, respectively) and *E. cloacae* isolates (susceptibility rates of 87%). The rate of tigecycline non-susceptibility was high (70% for *K. pneumoniae* and 50% for *Enterobacter* spp.), probably because of a strong selective pressure by this last-line antibiotic.

## Conclusion

This national survey on carbapenemase-producing isolates recovered in 2014 discovered a high rate of colistin resistance in *K. pneumoniae* and *E. cloacae* (6.2% and 7.7%, respectively) in France. These resistance rates remain much lower than those observed in other European countries such as Greece, Italy and Spain. No plasmid-encoded *mcr-1* gene was identified here. Therefore it seems that it is still possible to control the spread of those multidrug-resistant isolates based on accurate identification of colistin resistance and isolation of plasmid-encoded MCR-1 producers*.* Amikacin and fosfomycin remained the antibiotic agents most effective against those isolates which were resistant to polymyxins and produced a carbapenemase.
